# Genome-Wide Linkage Mapping for Preharvest Sprouting Resistance in Wheat Using 15K Single-Nucleotide Polymorphism Arrays

**DOI:** 10.3389/fpls.2021.749206

**Published:** 2021-10-14

**Authors:** Lingli Li, Yingjun Zhang, Yong Zhang, Ming Li, Dengan Xu, Xiuling Tian, Jie Song, Xumei Luo, Lina Xie, Desen Wang, Zhonghu He, Xianchun Xia, Yan Zhang, Shuanghe Cao

**Affiliations:** ^1^Institute of Crop Sciences, National Wheat Improvement Center, Chinese Academy of Agricultural Sciences, Beijing, China; ^2^Hebei Laboratory of Crop Genetics and Breeding, Institute of Cereal and Oil Crops, Hebei Academy of Agriculture and Forestry Sciences, Shijiazhuang, China; ^3^Shandong Provincial Key Laboratory of Dryland Farming Technology, College of Agronomy, Qingdao Agricultural University, Qingdao, China; ^4^International Maize and Wheat Improvement Center (CIMMYT) China Office, Beijing, China

**Keywords:** dormancy, KASP marker, QTL mapping, SNP chip, *Triticum aestivum*

## Abstract

Preharvest sprouting (PHS) significantly reduces grain yield and quality. Identification of genetic loci for PHS resistance will facilitate breeding sprouting-resistant wheat cultivars. In this study, we constructed a genetic map comprising 1,702 non-redundant markers in a recombinant inbred line (RIL) population derived from cross Yangxiaomai/Zhongyou9507 using the wheat 15K single-nucleotide polymorphism (SNP) assay. Four quantitative trait loci (QTL) for germination index (GI), a major indicator of PHS, were identified, explaining 4.6–18.5% of the phenotypic variances. Resistance alleles of *Qphs.caas-3AL, Qphs.caas-3DL*, and *Qphs.caas-7BL* were from Yangxiaomai, and Zhongyou9507 contributed a resistance allele in *Qphs.caas-4AL*. No epistatic effects were detected among the QTL, and combined resistance alleles significantly increased PHS resistance. Sequencing and linkage mapping showed that *Qphs.caas-3AL* and *Qphs.caas-3DL* corresponded to grain color genes *Tamyb10-A* and *Tamyb10-D*, respectively, whereas *Qphs.caas-4AL* and *Qphs.caas-7BL* were probably new QTL for PHS. We further developed cost-effective, high-throughput kompetitive allele-specific PCR (KASP) markers tightly linked to *Qphs.caas-4AL* and *Qphs.caas-7BL* and validated their association with GI in a test panel of cultivars. The resistance alleles at the *Qphs.caas-4AL* and *Qphs.caas-7BL* loci were present in 72.2 and 16.5% cultivars, respectively, suggesting that the former might be subjected to positive selection in wheat breeding. The findings provide not only genetic resources for PHS resistance but also breeding tools for marker-assisted selection.

## Introduction

Preharvest sprouting (PHS) refers to the germination of physiologically mature grains in spikes before harvest under rainy weather or humid environment (Groos et al., 2002). PHS is a major problem in cereal production and causes losses in seed vitality, yield, and quality (Xu et al., 2019). Wheat (*Triticum aestivum* L.) is one of the most important staple crops. The average annual loss of wheat caused by PHS exceeds $1 billion worldwide (Shao et al., 2018). Identification of genetic loci for PHS should be helpful for breeding resistant wheat cultivars.

Preharvest sprouting is a complex trait influenced by genetic and environmental factors (Barrero et al., 2015; Wang et al., 2019). Seed dormancy, an adaptive trait that prevents seeds from germinating, even under favorable conditions, is a major genetic factor for PHS (Née et al., 2017). Germination index (GI) is a common parameter to quantify genetic mechanisms underlying seed dormancy and PHS (Barrero et al., 2015). Some non-dormancy factors, such as spike erectness, spike and awn structure, and openness of florets, are also associated with PHS (Zhu et al., 2019).

Molecular markers have an important role in determining the genetic basis of agronomic traits in wheat (Collard and Mackill, 2008). Markers tightly linked with genes for PHS resistance can be used in marker-assisted selection (MAS). Using diverse mapping populations, many quantitative trait loci (QTL) for PHS resistance or seed dormancy on all 21 wheat chromosomes have been reported (Cao et al., 2016; Lin et al., 2016; Yang et al., 2019; Zhu et al., 2019; Tai et al., 2021). Among them, QTL on group 3 chromosomes and chromosome 4AL have major effects on PHS (Mori et al., 2005; Chen et al., 2008; Ogbonnaya et al., 2008; Shao et al., 2018; Vetch et al., 2019). A few genes for PHS in wheat were also isolated by map-based cloning. For example, *TaPHS1*, an *MFT* homolog, is the causal gene in *Qphs.pseru-3AS* (Liu et al., 2013; Jiang et al., 2018; Wang et al., 2020); *Tamyb10* genes at the *R* loci on chromosomes 3A, 3B, and 3D control grain coat color by regulating the accumulation of anthocyanins (Himi and Noda, 2005; Wang et al., 2016; Mares and Himi, 2021); *Mitogen-activated protein kinase kinase 3* (*MKK3*) is the causal gene of *Phs1-4AL* for seed dormancy in wheat (Torada et al., 2016; Martinez et al., 2020); and tandem duplicated plasma membrane protein genes (*PM19*) have been validated as candidates for a major dormancy QTL on chromosome 4AL through transcriptome analysis (Barrero et al., 2015; Shorinola et al., 2016). Homology-based cloning approaches were also used to identify PHS-related genes, such as *TaSdr* (Zhang et al., 2014, 2017), *Vp-1* (McCarty et al., 1991; Yang et al., 2007, 2013; Feng et al., 2017; Zhou et al., 2017), *Qsd1* (Sato et al., 2016; Onishi et al., 2017), and *DOG1* (Ashikawa et al., 2010; Nakabayashi et al., 2012; Rikiishi and Maekawa, 2014; Nishimura et al., 2018).

Yangxiaomai, a red-seeded Chinese landrace, has a high level of PHS resistance, whereas white-seeded Zhongyou9507 with good processing quality is susceptible to PHS. The objectives of this study are to mine QTL for PHS resistance in a recombinant inbred line (RIL) population derived from a Yangxiaomai/Zhongyou9507 cross and to develop breeding-friendly markers for selection of PHS-resistant varieties.

## Materials and Methods

### Plant Materials and Field Trials

The parents Yangxiaomai and Zhongyou9507 and 194 F_6_ RILs were planted at Beijing and Shijiazhuang (Hebei Province) in the 2011–2012 cropping season and at Gaoyi (Hebei Province) and Xinxiang (Henan Province) in the 2019–2020 cropping season. Field experiments were arranged in randomized complete blocks with three replications. Each plot was 1 m single row in which 30 seeds were sown. A panel of 101 wheat cultivars (Zhang et al., 2017) was used to determine the genetic effects of the QTL of interest.

### Evaluation of PHS Resistance

The GI was used as an indicator of PHS. Five spikes were harvested from each plot at physiological maturity characterized by loss of green color from the spike (Liu et al., 2013). The harvested spikes were air-dried for 2 days at room temperature, hand-threshed to avoid damage to embryos, and then stored in a refrigerator at −20°C to maintain dormancy until phenotyping (Zhang et al., 2017). Seeds were sterilized with 1% (V/V) of NaClO for 20 min, followed by three rinses with sterile water. Notably, 100 healthy seeds of each line were incubated in a 90 mm Petri dish containing a filter paper and 8 ml of distilled water at 20°C for 7 days. Germinated seeds were counted every day and removed. GI was calculated according to the following formula (Walker-Simmons, 1988): $GI=\frac{7\;\times \;n1\;+\;6\;\times \;n2\;+\;5\;\times \;n3\;+\;4\;\times \;n4\;+\ldots +\;1\;\times \;n7}{7\times total\;grains}\times 100$, where *n*_1_, *n*_2_, …, *n*_7_ are the number of seeds germinated on the first, second, and subsequent days until the seventh day.

### Statistical Analyses

Phenotypic correlation coefficients among environments, the best linear unbiased prediction (BLUP) values, ANOVA, and *t*-tests were carried out using SAS 9.4 software (SAS Institute Inc., Cary, NC, USA). Broad-sense heritability (*H*^2^) for PHS was calculated using the following formula: *H*^2^ $=\;\sigma_{\text{g}}^{2}$/($\sigma_{\text{g}}^{2}+\sigma_{\text{ge}}^{2}$/*e*+ $\sigma_{\text{ε}}^{2}$/*re*), where $\sigma_{\text{g}}^{2}$, $\sigma_{\text{ge}}^{2}$, and $\sigma_{\text{ε}}^{2}$ are the variances of genotype, genotype-environment interaction, and residual error, respectively, *r* is the number of replicates, and *e* is the number of environments (Nyquist and Baker, 1991).

### Genotyping and Linkage Map Construction

The 194 RILs and parents were genotyped with the wheat 15K single-nucleotide polymorphism (SNP) chips containing 13,947 SNP markers at China Golden Marker (Beijing) Biotech Co., Ltd. (http://www.cgmb.com.cn/). To reduce the impact of low-quality SNPs on mapping results, SNP data were processed as follows: (1) Heterozygous loci were treated as missing data, and (2) SNPs with low minor allele frequencies (<0.3) and missing values (>0.2) were excluded using Tassel version 5.0 (Bradbury et al., 2007). Redundant markers were eliminated by the BIN function in QTL IciMapping version 4.2 (Meng et al., 2015). Joinmap version 4.0 was used for linkage map construction (Stam, 1993), and genetic distances between markers were calculated according to the Kosambi mapping function (Kosambi, 1943).

### QTL Analysis

Composite interval mapping (CIM) was used to search QTL of phenotypic traits from each environment and BLUP value by Windows QTL Cartographer version 2.5 (Zeng, 1994; Wang et al., 2012). Significant QTLs were identified if the logarithm of odds (LOD) values were more than the threshold of 2.5 (Yan et al., 2006). According to International Wheat Genome Sequencing Consortium (IWGSC) RefSeq 1.0 [(International Wheat Genome Sequencing Consortium (IWGSC), 2018) http://plants.ensembl.org/index.html], the physical positions of QTL were figured out by the closely linked flanking markers. The genetic maps of QTL were drawn using MapChart version 2.3 (Voorrips, 2002). The analysis of epistatic effects among the QTL was performed using IciMapping version 4.2.

### KASP Marker Development and Validation

Kompetitive allele-specific PCR primers (Supplementary Table 5) were designed using PolyMarker (Ramirez-Gonzalez et al., 2015). Primer mixture was prepared with 46 μl of H_2_O, 30 μl of common primer (100 μM), and 12 μl of each tailed primer (100 μM). PCR was performed in a 384-well plate, and each reaction of ~3 μl comprising 20–30 ng of genomic DNA, 1.5 μl of 2 × KASP master mix (V4.0, LGC Genomics, Hoddesdon, UK), 0.0336 μl of primer mixture, and 1.5 μl of H_2_O. Thermal cycling profile of PCR consisted of hot start at 95°C for 15 min, 10 touchdown cycles (95°C for 20 s and touchdown at 65 and −1°C per cycle for 25 s), and followed by 35 additional cycles (95°C for 20 s and 57°C for 60 s). The 384-well optically clear plates were read on PHERAstarplus SNP (BMG Labtech GmbH, Ortenberg, Germany), and data analysis was carried out using KlusterCaller (LGC, Hoddesdon, UK).

## Results

### Phenotypic Evaluation

The parents Yangxiaomai and Zhongyou9507 and RILs were evaluated for PHS resistance in four environments. The phenotypes of seed germination in parents Yangxiaomai and Zhongyou9507 were depicted in Supplementary Figure 1. Yangxiaomai had a significantly lower GI (4.3%) than Zhongyou9507 (72.3%) across environments (Supplementary Figure 2). GI for the RIL population showed continuous variation, indicating polygenic inheritance (Supplementary Figure 2). The GI frequencies were skewed toward resistance, suggesting the presence of major genetic loci. GI was significantly correlated among environments with correlation coefficients of 0.53–0.73 (Supplementary Table 1). ANOVA indicated that genotypes and environments, as well as their interactions, had significant effects on GI (Supplementary Table 2). The broad-sense heritability of GI was high (0.88) across environments, denoting that GI variation was mainly determined by genotypes.

### Linkage Map Construction and QTL Analysis

The RIL population was genotyped by 15K SNP chips, and 4,515 polymorphic markers were used to construct a genetic map with 1,702 bin markers, spanning 2,630.9 cM on 21 wheat chromosomes (Supplementary Table 3 and Supplementary Figure 3). The average linkage group was 125.3 cM with an average marker interval of 1.6 cM. Overall, 1,743 (38.6%), 1,750 (38.8%), and 1,022 (22.6%) markers were mapped to the A, B, and D sub-genomes with average marker densities of 1.5, 1.2, and 2.2 cM, respectively (Supplementary Table 3 and Supplementary Figure 3).

**Figure 1:**
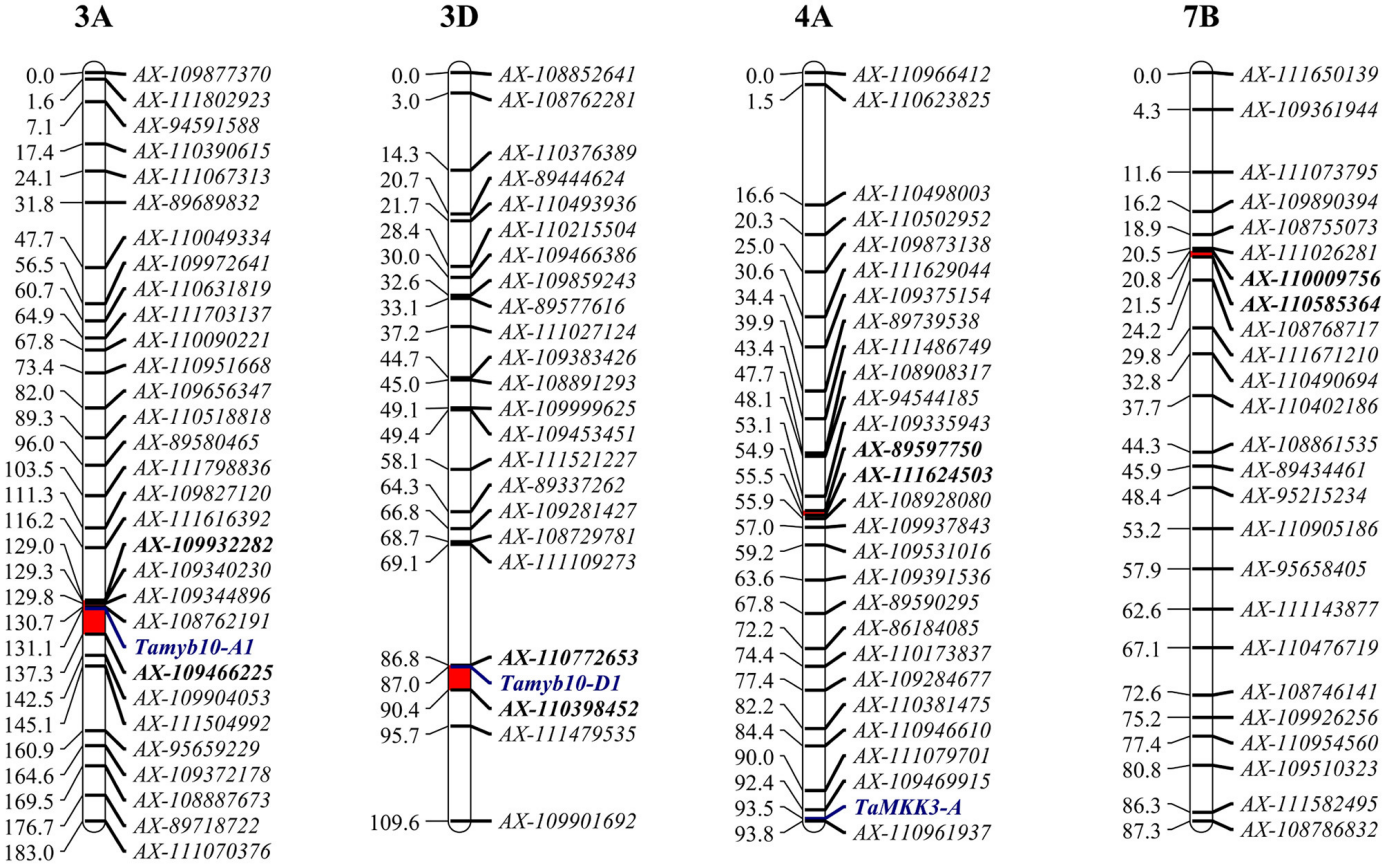
Genetic mapping of *Qphs.caas-3AL, Qphs.caas-3DL, Qphs.caas-4AL*, and *Qphs.caas-7BL* in the Yangxiaomai/Zhongyou9507 recombinant inbred line (RIL) population. Target regions of the quantitative trait loci (QTL) are indicated as red bars; gene-specific markers are shown in blue script; and flanking markers are shown in bold.

Four QTLs for PHS were detected by CIM on the linkage groups 3AL (*Qphs.caas-3AL*), 3DL (*Qphs.caas-3DL*), 4AL (*Qphs.caas-4AL*), and 7BL (*Qphs.caas-7BL*) (Table 1 and Figure 1). Alleles for resistance to PHS on chromosome arms 3AL, 3DL, and 7BL loci were from Yangxiaomai, whereas the resistance allele on 4AL was contributed by Zhongyou9507. *Qphs.caas-3DL* was identified across all four environments and explained 8.9–18.5% of the phenotypic variances; *Qphs.caas-3AL* and *Qphs.caas-4AL* were detected in three of four environments, explaining 10.5–13.5 and 4.6–10.6% of the phenotypic variances, respectively; and *Qphs.caas-7BL* accounting for 5.0–6.7% of the phenotypic variances was detected in two environments.

**Table 1:** QTL for GI detected in the Yangxiaomai/Zhongyou9507 RIL population.

**QTL**	**Environment**	**Flanking marker**	**Physical interval (Mb)**	**LOD^a^**	**PVE (%)^b^**	**Add^c^**
*Qphs.caas-3AL*	2020 XX	*AX-109932282-AX-109340230*	700.4–702.5	6.3	10.5	4.7
	2020 GY	*AX-108762191-AX-109466225*	703.9–709.2	7.5	13.5	6.7
	2012 SJZ	*AX-109932282-AX-109340230*	700.4–702.5	6.5	11.2	5.4
	BLUP	*AX-109932282-AX-109340230*	700.4–702.5	6.8	10.6	3.6
*Qphs.caas-3DL*	2020 XX	*AX-110772653-AX-110398452*	570.2–575.1	7.1	12.2	5.1
	2020 GY	*AX-110772653-AX-110398452*	570.2–575.1	10.8	18.5	7.9
	2012 SJZ	*AX-110772653-AX-110398452*	570.2–575.1	7.3	13.4	6.1
	2012 BJ	*AX-110772653-AX-110398452*	570.2–575.1	4.9	8.9	6.6
	BLUP	*AX-110772653-AX-110398452*	570.2–575.1	8.7	14.4	4.2
*Qphs.caas-4AL*	2020 XX	*AX-89597750-AX- 111624503*	489.1–532.2	3.3	5.2	−3.3
	2020 GY	*AX-89597750-AX- 111624503*	489.1–532.2	3.0	4.6	−3.8
	2012 BJ	*AX-89597750-AX- 111624503*	489.1–532.2	6.1	10.6	−7.1
	BLUP	*AX-89597750-AX- 111624503*	489.1–532.2	5.4	8.2	−3.2
*Qphs.caas-7BL*	2020 GY	*AX-110009756-AX-110585364*	522.6–529.7	3.7	5.7	4.3
	2012 BJ	*AX-110009756-AX-110585364*	522.6–529.7	3.5	6.1	5.4
	BLUP	*AX-110009756-AX-110585364*	522.6–529.7	3.2	5.0	2.4

a *A LOD threshold of 2.5 was used for the declaration of QTL*.

b *Percentage of phenotypic variances explained by QTL*.

c *Positive and negative additive effects indicated increasing effects from Yangxiaomai and Zhongyou9507, respectively*.

*QTL, quantitative trait loci; GI, germination index; RIL, recombinant inbred line; XX, Xinxiang; GY, Gaoyi; SJZ, Shijiazhuang; BJ, Beijing; LOD, logarithm of odds*.

### Combinational Effects of the Stable QTL for PHS Resistance

**Figure 2:**
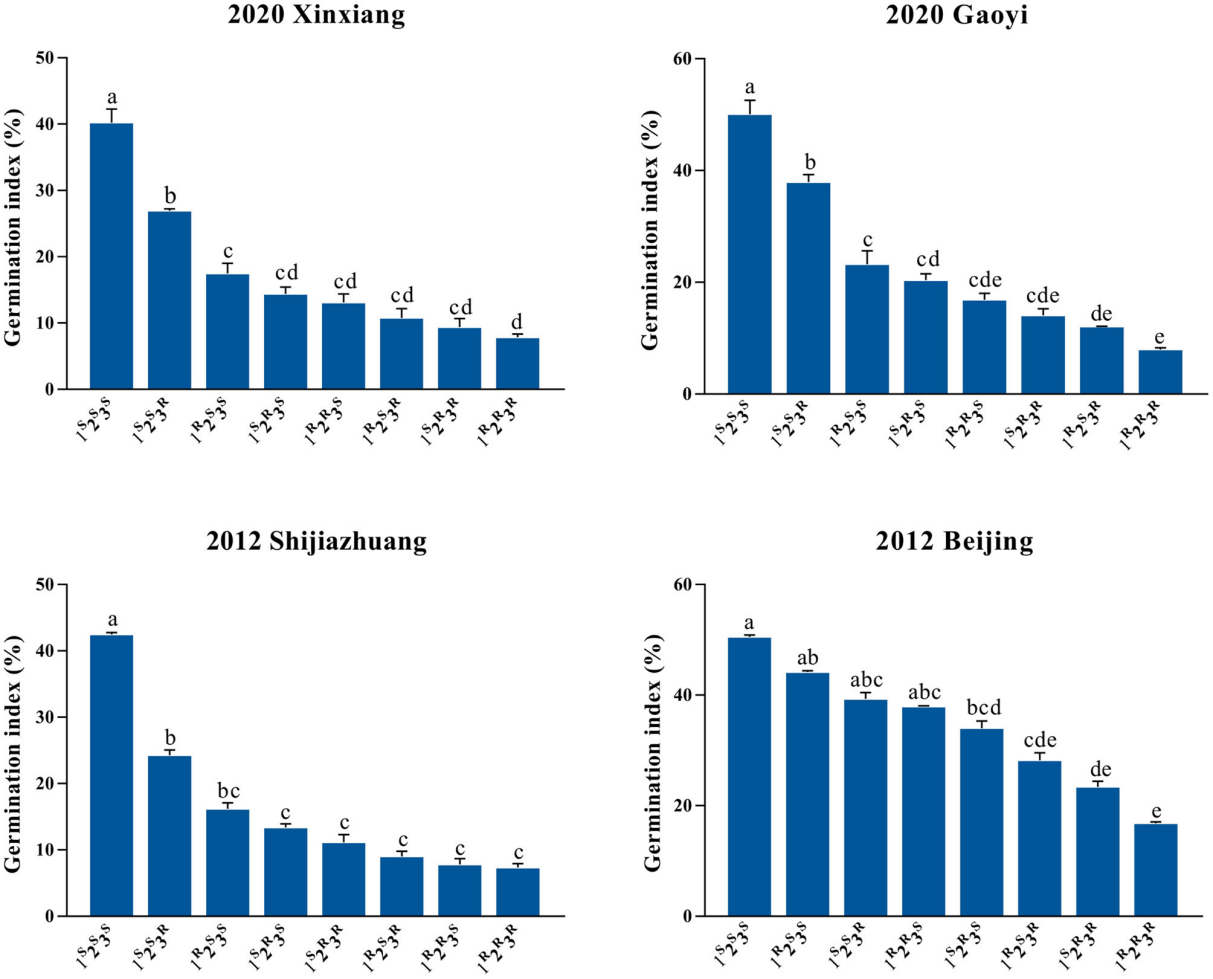
Distributions of germination index (GI) among eight genotypic combinations of *Qphs.caas-3AL, Qphs.caas-3DL*, and *Qphs.caas-4AL* grown in four environments. The *x*-axis shows the genotypic groups, and the *y*-axis indicates GI (%). The numbers 1, 2, and 3 represent *Qphs.caas-3AL, Qphs.caas-3DL*, and *Qphs.caas-4AL*, respectively; the superscript letters R and S represent resistance and susceptible alleles, respectively. Genotypes with different letters indicate significant differences (*P* < 0.05) in GI, and those with the same letters show no significant differences (*P* > 0.05). Error bar, standard error of each group mean.

Quantitative trait loci for a given trait detected in more than one-half of tested environments can be considered stable genetic loci (Cao et al., 2020). The QTL *Qphs.caas-3AL, Qphs.caas-3DL*, and *Qphs.caas-4AL* fulfilled that criterion. To confirm their genetic effects on PHS, the population was classified into eight groups based on the closest flanking SNPs for each QTL (Supplementary Table 4). *Qphs.caas-3AL, Qphs.caas-3DL*, and *Qphs.caas-4AL* were temporarily designated as the loci *1, 2*, and *3*, respectively, and R and S represented resistance and susceptible alleles, respectively. The GI values of eight groups (i.e., *1*^*R*^*2*^*R*^*3*^*R*^, *1*^*R*^*2*^*R*^*3*^*S*^, *1*^*R*^*2*^*S*^*3*^*R*^, *1*^*S*^*2*^*R*^*3*^*R*^, *1*^*R*^*2*^*S*^*3*^*S*^, *1*^*S*^*2*^*R*^*3*^*S*^, *1*^*S*^*2*^*S*^*3*^*R*^, and *1*^*S*^*2*^*S*^*3*^*S*^) were compared across four environments (Figure 2 and Supplementary Table 4).

The eight groups were ranked according to the GI in four environments and BLUP values. In general, more resistance alleles conferred lower GI, demonstrating cumulative effects of resistance alleles at the three loci (Figure 2). RILs with genotype *1*^*R*^*2*^*R*^*3*^*R*^ had the lowest GI, and those with *1*^*S*^*2*^*S*^*3*^*S*^ exhibited the highest GI across all environments. However, the GI of RILs with *1*^*R*^*2*^*R*^*3*^*S*^ were higher than those with *1*^*S*^*2*^*R*^*3*^*S*^ in Beijing 2012, suggesting that the genetic effect of locus *1, Qphs.caas-3AL*, was significantly affected by the environment in some cases. No epistatic effects were detected among the QTL. Regression analysis also showed that the lines carrying more resistance alleles had higher PHS resistance in individual environments and the BLUP data (Supplementary Figure 4). Thus, the pyramiding of resistance alleles was effective in improving PHS resistance.

### Relationship of *Qphs.caas*-3AL and *Qphs.caas*-3DL With Tamyb10

According to IWGSC RefSeq 1.0, *Qphs.caas-3AL* and *Qphs.caas-3DL* were delimited in the intervals of 700.4–709.2 Mb and 570.2–575.1 Mb, respectively, based on their flanking markers. This placed PHS-related genes *Tamyb10-A1* (~703.9 Mb) and *Tamyb10-D1* (~570.8 Mb), in the regions of *Qphs.caas-3AL* and *Qphs.caas-3DL*, respectively (Himi et al., 2011). To confirm the genetic relationship of the two genes with *Qphs.caas-3AL* and *Qphs.caas-3DL*, we re-genotyped the Yangxiaomai/Zhongyou9507 RIL population using a KASP marker for *Tamyb10-A1* and an STS marker for *Tamyb10-D1* (Supplementary Table 5) (Himi et al., 2011; Wang et al., 2016). In these analyses, *Tamyb10-A1* and *Tamyb10-D1* were mapped to the genetic regions of *Qphs.caas-3AL* and *Qphs.caas-3DL*, respectively (Figure 1). Thus, *Tamyb10-A1* and *Tamyb10-D1* were likely causal genes in *Qphs.caas-3AL* and *Qphs.caas-3DL*, respectively.

### Relationship Between *Qphs.caas-4AL* and Reported PHS Resistance Genes on Chromosome 4AL

*TaMKK3-A* was reported as a major gene controlling seed dormancy on chromosome 4AL (Torada et al., 2016). Based on the IWGSC RefSeq 1.0, *TaMKK3-A* is located at the site of ~609.1 Mb (GenBank accession number: LC091368.1) (Liton et al., 2021). *Qphs.caas-4AL* spans the interval of 489.1–532.2 Mb according to flanking markers *AX-89597750* and *AX-111624503*, suggesting that *TaMKK3-A* is not in the target region of *Qphs.caas-4AL*. We sequenced the *TaMKK3-A* gene in Yangxiaomai and Zhongyou9507 to confirm the relationship between *TaMKK3-A* and *Qphs.caas-4AL*. Sequence analysis showed that *TaMKK3-A* had no polymorphic sites between two parents in all exons, but three SNPs were detected in the introns (Supplementary Figure 5). A KASP marker *KASP-6464* was developed for *TaMKK3-A*. Linkage mapping showed that *KASP-6464* was out of the target region of *Qphs.caas-4AL* (Figure 1). Association analysis also indicated that *TaMKK3-A* had no significant effect on GI in three environments (Supplementary Table 6). Therefore, *TaMKK3-A* was not a candidate gene in *Qphs.caas-4AL*.

*PM19-A1* is a second PHS-related gene in chromosome 4AL. However, it is positioned at ~604.1 Mb, which is far from *Qphs.caas-4AL* (489.1–532.2 Mb) according to the IWGSC RefSeq 1.0. We also compared the sequences of *PM19-A1* between Yangxiaomai and Zhongyou9507 and found no polymorphic sites in the open reading frame and the previously reported 18 bp indel in the promoter (Barrero et al., 2015; Shorinola et al., 2016, 2017). Therefore, *PM19-A1* was not the causal gene in *Qphs.caas-4AL*.

No genes related to PHS were isolated on 7B so far, so no candidate genes could be used to perform sequencing and genetic mapping analyses for *Qphs.caas-7BL*. *Qphs.caas-7BL* is defined in the interval of 522.6–529.7 Mb based on its flanking markers according to IWGSC RefSeq 1.0. QTL related to PHS, which have been reported on 7B, were summarized in Supplementary Table 7. The location of *Qphs.caas-7BL* is different from those of the previously reported QTL based on their flanking markers.

### Genetic Effects of *Qphs.caas-4AL* and *Qphs.caas-7BL* on GI in a Panel of Cultivars

The causal genes of *Qphs.caas-4AL* and *Qphs.caas-7BL* remained unknown although they could explain 4.6–10.6% of the phenotypic variances. To further decipher the importance of *Qphs.caas-4AL* and *Qphs.caas-7BL*, we attempted to identify their genetic effects in a panel of cultivars. An SNP *AX-89597750* closely linked with *Qphs.caas-4AL* was converted to a KASP marker. The KASP marker was mapped at the position of *AX-89597750*, indicating that it could act as a closely linked marker of *Qphs.caas-4AL*. We detected the genetic effect of *Qphs.caas-4AL* on GI using the KASP marker in the cultivar panel (Supplementary Table 8). Genotypes with the resistance allele had significantly lower GI than those with the susceptible allele (Table 2). Moreover, a majority of genotypes (72.2%) possessed the resistance allele of *Qphs.caas-4AL*, suggesting that it had been subjected to selection in wheat breeding (Table 2). Another KASP marker was developed based on SNP *AX-9496498* closely linked to *Qphs.caas-7BL*. The KASP marker was mapped to the target region of *Qphs.caas-7BL* in the mapping population. Like *Qphs.caas-4AL, Qphs.caas-7BL* was also significantly associated with PHS resistance (Table 2), but only 16.5% of cultivars carried the resistance allele.

**Table 2:** The effects of *Qphs.caas-4AL* and *Qphs.caas-7BL* on GI in a natural population.

**QTL**	**Genotype**	**Number**	**GI (%)**	***P*-value**
*Qphs.caas-4AL*	AA	70	31.82	0.03^*^
	BB	27	40.95	
*Qphs.caas-7BL*	AA	81	36.73	0.008^**^
	BB	16	23.47	

* *P < 0.05;*

** *P < 0.01*.

*The wheat lines in the natural population are listed in Supplementary Table 8*.

*GI, germination index; QTL, quantitative trait loci; AA, the allele from Zhongyou9507; BB, the allele fromYangxiaomai*.

## Discussion

### Grain Color Is a Major Factor Modulating PHS

The red-grain Yangxiaomai and white-grain Zhongyou9507 have a relatively low and higher GI, respectively. In this study, we confirmed that *Qphs.caas-3AL* and *Qphs.caas-3DL* co-localized with *Tamyb10-A1* and *Tamyb10-D1*, respectively, at the *R* loci for grain color (Himi et al., 2011; Lang et al., 2021; Mares and Himi, 2021). Wang et al. (2016) also observed that *Tamyb10-D1* was significantly (*P* < 0.001) associated with GI in a natural population. Thus, grain color is probably regulated by *Tamyb10* alleles in *Qphs.caas-3AL* and *Qphs.caas-3DL*, which also cause significant differences in GI between Yangxiaomai and Zhongyou9507. Pleiotropic QTL for grain color and PHS resistance on chromosomes 3AL and 3DL were identified in a genome-wide association study conducted by Lin et al. (2016). These findings also confirm that grain color has a great effect on PHS resistance in wheat breeding.

### *Qphs.caas-4AL* Has Potential Value for Wheat Breeding

*Qphs.caas-4AL*, as a stable QTL, accounted for 4.6–10.6% of the phenotypic variances. Although quite a few QTL for wheat PHS resistance on chromosome 4A have also been reported (Kato et al., 2001; Mares et al., 2005; Torada et al., 2005; Imtiaz et al., 2008; Rasul et al., 2009; Singh et al., 2010; Kulwal et al., 2012; Cabral et al., 2014; Jiang et al., 2015; Cao et al., 2016; Zhou et al., 2017; Martinez et al., 2018; Zuo et al., 2019; Liton et al., 2021), *Qphs.caas-4AL* appears to be unique based on genetic mapping and physical locations of the flanking SNPs according to IWGSC RefSeq 1.0 (Supplementary Table 9). QTL pyramiding plays an important role in breeding, and resistance allele combinations of QTL for PHS have been reported previously (Imtiaz et al., 2008; Shao et al., 2018; Liton et al., 2021). In this study, we identified that the RILs combining resistance alleles in *Qphs.caas-3AL, Qphs.caas-3DL*, and *Qphs.caas-4AL* had the lowest GI (Figure 2). *Qphs.caas-4AL* also improved resistance to PHS in the absence of the alleles for red-grain color (Figure 2 and Supplementary Table 4). This indicated that *Qphs.caas-4AL* could function independently of grain color. We converted an SNP tightly linked to *Qphs.caas-4AL* into a KASP marker. Genotyping by the KASP marker showed that most of the cultivars (72.2%) carried the resistance allele in *Qphs.caas-4AL* in the test panel (Table 2), indicating that the resistance allele of *Qphs.caas-4AL* might undergo positive selection in breeding programs. *Qphs.caas-4AL* is also significantly associated with GI (Table 2). Thus, the KASP marker will be useful for MAS to improve PHS tolerance in wheat. Grain color is an important parameter for wheat appearance quality. Red-grain wheat usually has high resistance to PHS but is adverse to make Chinese traditional food, such as steamed bread and noodles (Fakthongphan et al., 2016; Shao et al., 2018). Thus, *Qphs.caas-4AL* is a better choice for improvement of PHS than *Qphs.caas-3AL* and *Qphs.caas-3DL* at least in Chinese wheat breeding. Overall, these findings indicate that *Qphs.caas-4AL* is a valuable genetic locus for PHS in wheat breeding.

## Data Availability Statement

The original contributions presented in the study are included in the article/Supplementary Materials, further inquiries can be directed to the corresponding authors.

## Author Contributions

LL and SC wrote the manuscript. LL, YiZ, ML, DX, XT, JS, and XL performed the experiments. SC and LL analyzed the data. YoZ, LX, and DW participated in the field trials. SC and YaZ designed the experiments. XX and ZH assisted in writing the manuscript. All authors read and approved the final manuscript.

## Funding

This study was funded by the National Natural Science Foundation of China (Grant Nos. 91935304 and 31571663), the National Key Research and Development Programs of China (Grant No. 2016YFD0100502), and the CAAS Science and Technology Innovation Program.

## Conflict of Interest

The authors declare that the research was conducted in the absence of any commercial or financial relationships that could be construed as a potential conflict of interest.

## Publisher's Note

All claims expressed in this article are solely those of the authors and do not necessarily represent those of their affiliated organizations, or those of the publisher, the editors and the reviewers. Any product that may be evaluated in this article, or claim that may be made by its manufacturer, is not guaranteed or endorsed by the publisher.

## Supplementary Material

Supplementary Figure 1 Seed germination of Yangxiaomai and Zhongyou9507. YXM, Yangxiaomai; ZY9507, Zhongyou9507.

Supplementary Figure 2 Distributions of GI in the Yangxiaomai/Zhongyou9507 RIL population in four environments. Black arrows point to the GI of the parents. GI, germination index; RIL, recombinant inbred line; YXM, Yangxiaomai; ZY9507, Zhongyou9507.

Supplementary Figure 3 Genetic map of the Yangxiaomai/Zhongyou9507 RIL population determined from 15K SNP arrays. RIL, recombinant inbred line; SNP, single-nucleotide polymorphism.

Supplementary Figure 4 Linear regression between the resistance allele number of the QTL and GI in four environments and BLUP values in the Yangxiaomai/Zhongyou9507 population. The QTL include *Qphs.caas-3AL, Qphs.caas-3DL, Qphs.caas-4AL*, and *Qphs.caas-7BL*. QTL, quantitative trait loci; GI, germination index; BLUP, best linear unbiased prediction.

Supplementary Figure 5 Schematic of the open reading frame of *TaMKK3-A* and its polymorphic sites between Yangxiaomai (YXM) and Zhongyou9507 (ZY9507). Bold bars and thin lines indicate exons and introns, respectively.

Supplementary Table 1 Correlation coefficients of GI from the Yangxiaomai/Zhongyou9507 RIL population among four environments.

Supplementary Table 2 Variance analysis for GI of the Yangxiaomai/Zhongyou9507 RIL population.

Supplementary Table 3 Information of genetic map construction for Yangxiaomai/Zhongyou9507 RIL population using 15K SNP chips.

Supplementary Table 4 GI of eight genotypic groups defined by three stable QTL in the Yangxiaomai/Zhongyou9507 RIL population.

Supplementary Table 5 Primers used in this study.

Supplementary Table 6 Association analysis of TaMKK3-A and GI in the Yangxiaomai/Zhongyou9507 RIL population.

Supplementary Table 7 Information of reported QTL for PHS on the chromosome 7B.

Supplementary Table 8 Genotypes of Qphs.caas-4AL and Qphs.caas-7BL and GI of 101 wheat cultivars.

Supplementary Table 9 Information of reported QTL for PHS on the chromosome 4A.
