# Supplementary figures and images for: Genome-Wide Linkage Mapping for Preharvest Sprouting Resistance in Wheat Using 15K Single-Nucleotide Polymorphism Arrays
Source: Front Plant Sci. 2021 Oct 14;12:749206. doi: 10.3389/fpls.2021.749206 (PMC8551680; doi:10.3389/fpls.2021.749206)

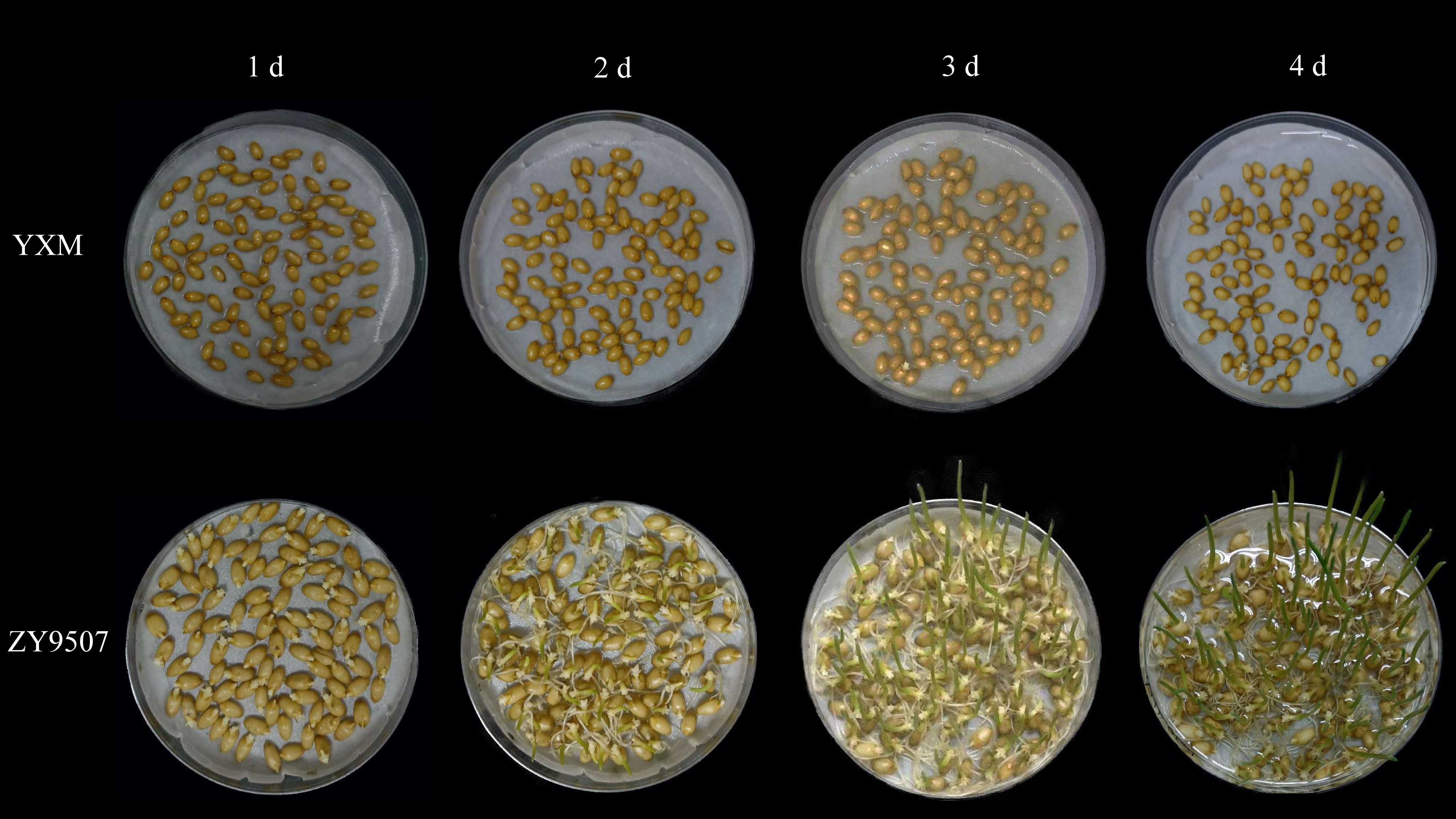

Supplement: Supplementary Figure 1 — Seed germination of Yangxiaomai and Zhongyou9507. YXM, Yangxiaomai; ZY9507, Zhongyou9507. [file Image_1.TIF]

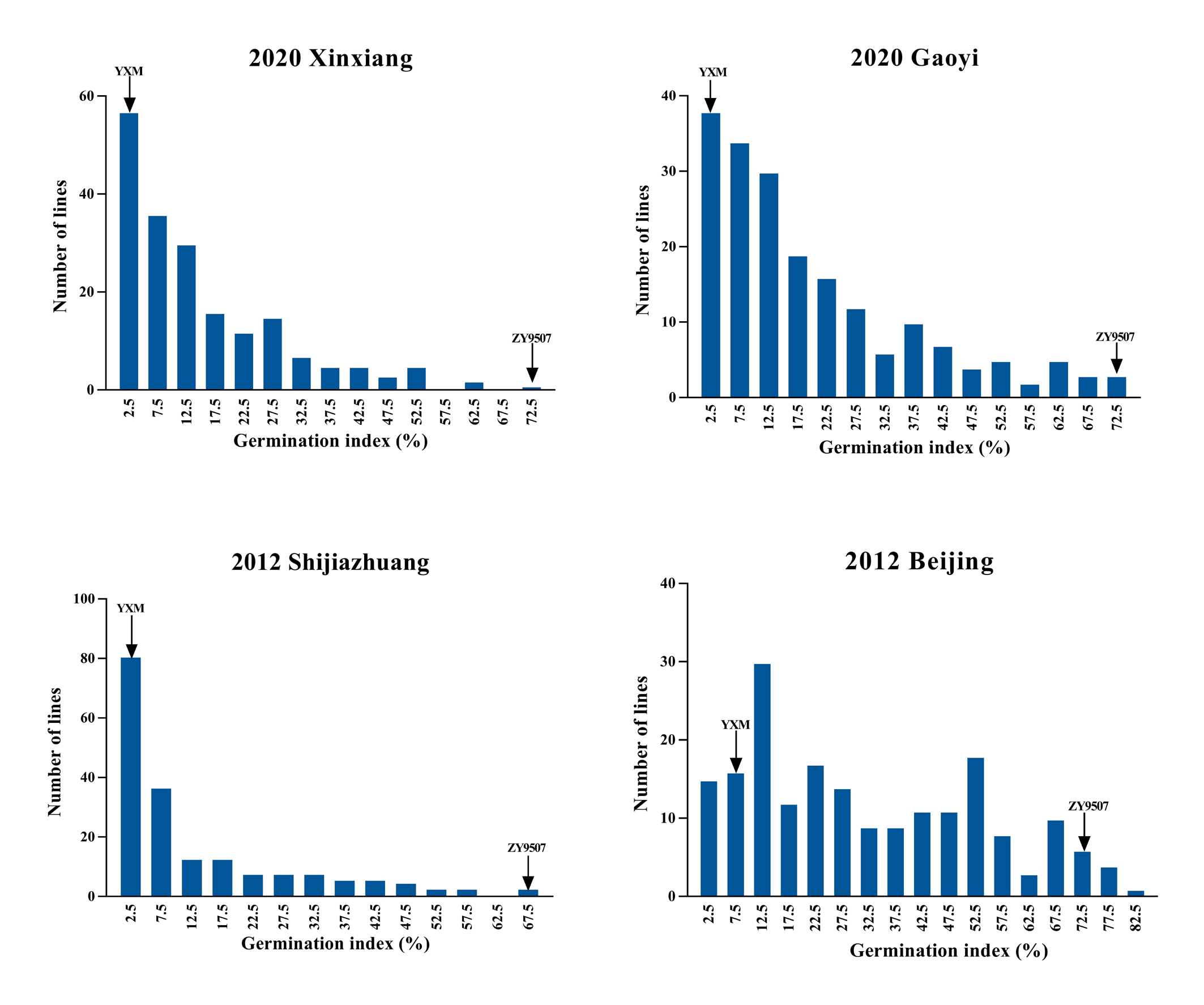

Supplement: Supplementary Figure 2 — Distributions of GI in the Yangxiaomai/Zhongyou9507 RIL population in four environments. Black arrows point to the GI of the parents. GI, germination index; RIL, recombinant inbred line; YXM, Yangxiaomai; ZY9507, Zhongyou9507. [file Image_2.TIF]

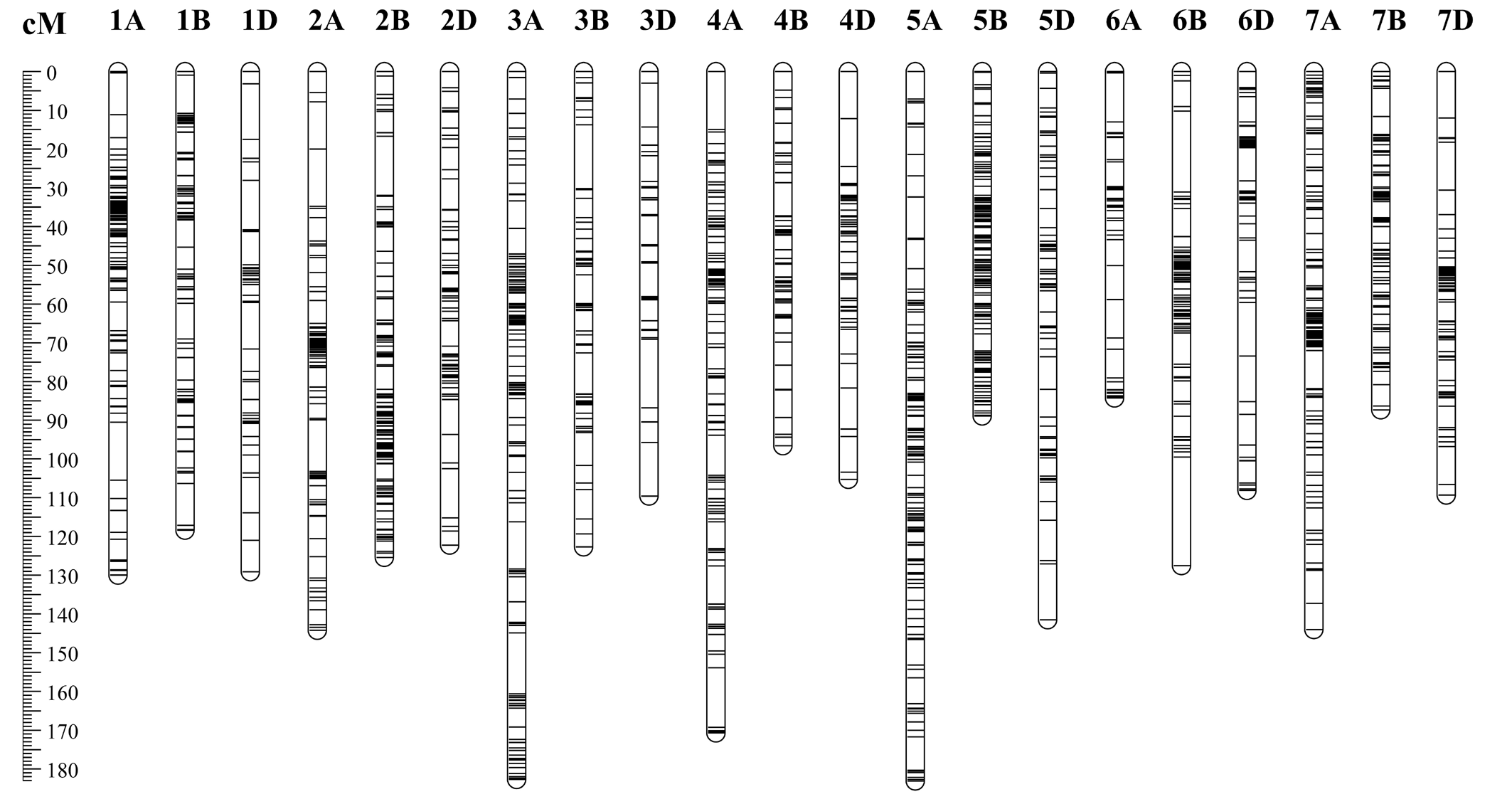

Supplement: Supplementary Figure 3 — Genetic map of the Yangxiaomai/Zhongyou9507 RIL population determined from 15K SNP arrays. RIL, recombinant inbred line; SNP, single-nucleotide polymorphism. [file Image_3.TIF]

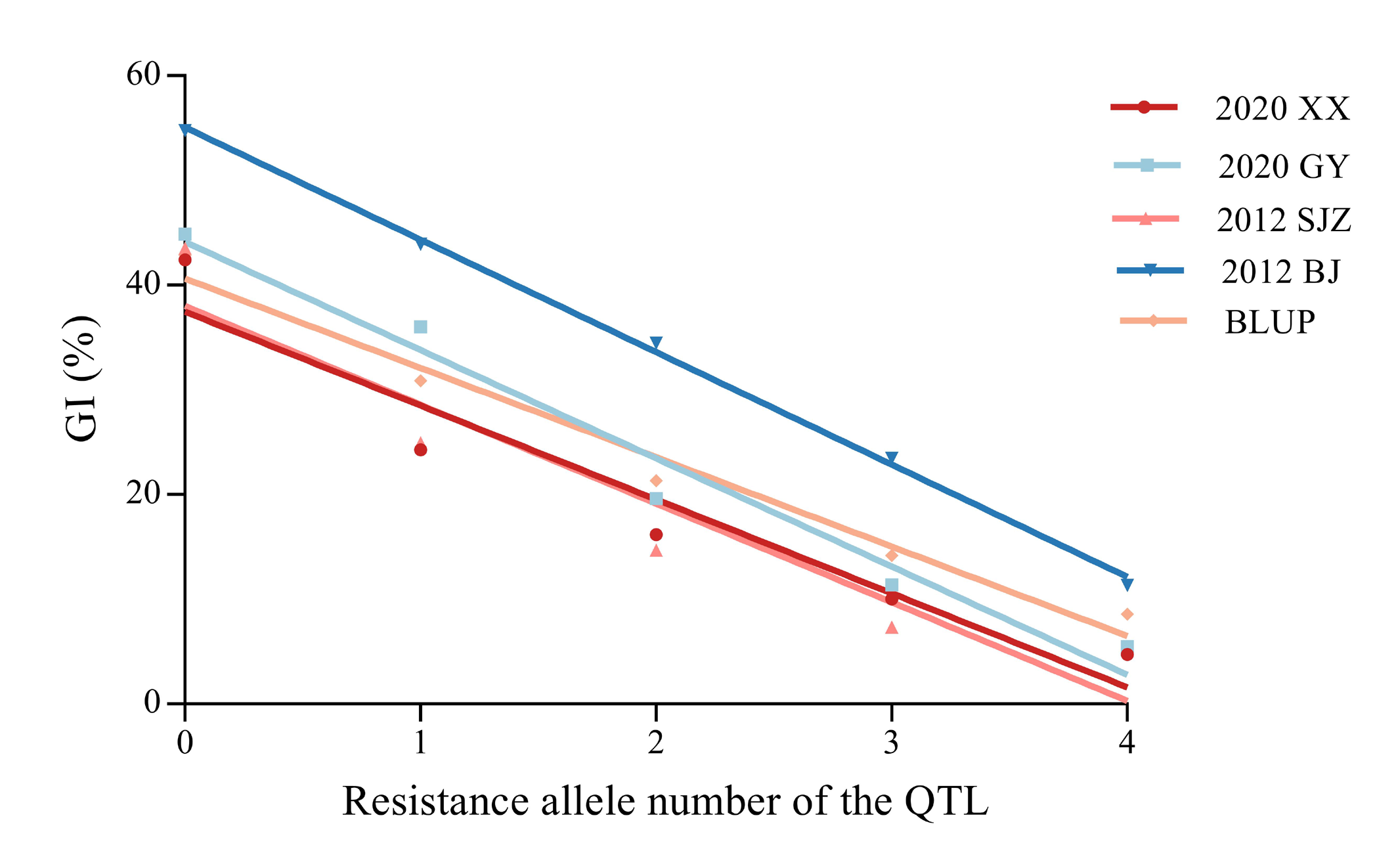

Supplement: Supplementary Figure 4 — Linear regression between the resistance allele number of the QTL and GI in four environments and BLUP values in the Yangxiaomai/Zhongyou9507 population. The QTL include Qphs.caas-3AL, Qphs.caas-3DL, Qphs.caas-4AL, and Qphs.caas-7BL. QTL, quantitative trait loci; GI, germination index; BLUP, best linear unbiased prediction. [file Image_4.TIF]

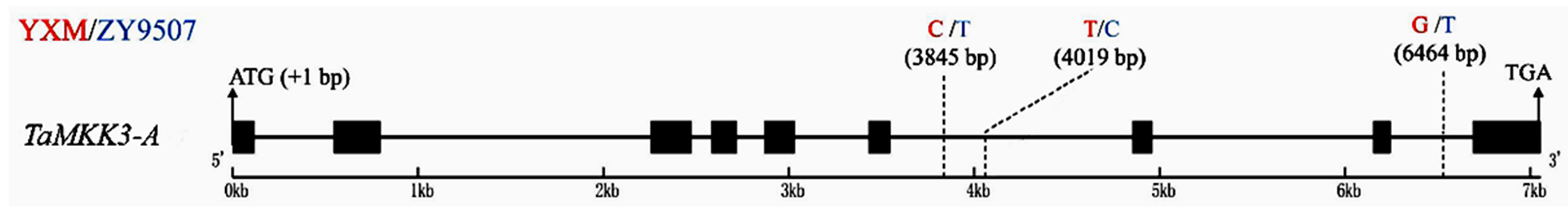

Supplement: Supplementary Figure 5 — Schematic of the open reading frame of TaMKK3-A and its polymorphic sites between Yangxiaomai (YXM) and Zhongyou9507 (ZY9507). Bold bars and thin lines indicate exons and introns, respectively. [file Image_5.TIF]
